# Free Light Chains, High Mobility Group Box 1, and Mortality in Hemodialysis Patients

**DOI:** 10.3390/jcm11236904

**Published:** 2022-11-23

**Authors:** Antonio Lacquaniti, Susanna Campo, Giuseppe Falliti, Daniele Caruso, Romana Gargano, Elena Giunta, Paolo Monardo

**Affiliations:** 1 Nephrology and Dialysis Unit, Papardo Hospital, 98158 Messina, Italy; 2 Clinical Pathology Unit, Papardo Hospital, 98158 Messina, Italy; 3 Department of Economics, University of Messina, 98122 Messina, Italy; 4 Microbiology and Virology Unit, Papardo Hospital, 98158 Messina, Italy

**Keywords:** uremic toxin, free light chains, HMGB1, CD4+/CD8+ ratio, hemodialysis

## Abstract

Background: Uremic toxins are associated with immune dysfunction and inflammation. The inadequate removal by hemodialysis (HD) of serum free light chains (FLCs) determines their accumulation. This study evaluated FLCs in HD patients, analyzing their relations with other biomarkers, such as serum high mobility group box 1 (HMGB1). Methods: FLC and HMGB1 were evaluated in a cohort of 119 HD patients. κFLC and λFLC were summated to give a combined (c) FLC concentration. Patients were followed prospectively until the end of the observation period of four years, or until the endpoint: the patient’s death. Results: cFLC values in HD patients were 244.4 (197.9–273.5) mg/L. We detected a significant reduction in CD8+ cells and a decreased CD4+/CD8+ ratio. HMGB1 levels were 94.5 (55–302) pg/mL. After multivariate analysis, cFLCs correlated with β2-microglobulin and the CD4+/CD8+ ratio. Subjects with cFLC values above 263 mg/L and with sHMGB1 values < 80 pg/mL experienced a significantly faster evolution to the endpoint (mean follow-up time to progression of 27.5 and 28.5 months, respectively; *p* < 0.001). After an adjusted multivariate Cox analysis, cFLCs were associated with 11% increased risk of death, whereas low sHMGB1 increased this risk by 5%. Conclusions: cFLCs and HMGB1 reflect the inflammation and immune dysfunction in HD patients representing two strong and independent risk markers of mortality.

## 1. Introduction

Uremic toxins represent independent risk factors for mortality in end-stage renal disease (ESRD) [1,2]; these substances, poorly removed by diffusive hemodialysis (HD) techniques, are associated with the pathological features of uremia, such as immune dysfunction, inflammation, and adverse cardiovascular outcomes [3,4,5,6]. Whereas systemic inflammation contributes to atherosclerosis, cardiovascular disease, and anemia, immune deficiency leads to an impaired response to vaccination and an increased incidence and severity of microbial infections [7]. These two entities, not mutually exclusive, could represent two sides of the same coin [8]: uremic-associated inflammation is closely related to the activation of the innate immune system and the depletion and impaired activities of T and B lymphocytes [9,10].

High mobility group box 1 (HMGB1), belonging to the danger-/damage-associated molecular patterns (DAMPs), is produced by these defensive immune cells, triggering an innate immune response by activating the Toll-like receptors [11,12]. Various studies assessed high HMGB1 levels in nephropathic patients [13,14,15,16].

High levels of serum HMGB1 characterized ESRD patients treated by chronic HD or peritoneal dialysis, positively correlating with pro-inflammatory cytokines and related with complications, such as heart failure and arteriovenous fistula occlusion [17].

This molecule was also evaluated in blood and peritoneal dialysis (PD) effluence in adult and pediatric PD patients with acute clinical peritonitis. A significant elevation of HMGB1 distinguished these patients with a gradual decline in its values during effective antibiotic treatments, suggesting a diagnosis and prognostic properties [18,19].

Similarly, high HMGB1 levels involved septic patients who developed acute kidney injury (AKI) [20].

Moreover, pro- and anti-inflammatory mediators, as well as DAMPs, play important roles in regulating the immunological response that mediates the severity and complications of sepsis, and the continuous veno-venous hemofiltration and selective hemofilters may assist in reducing acute inflammation through the removal of pro-inflammatory cytokines and signaling molecules. However, AKI patients with a high HMGB1 clearance rate by hemofilter were associated with a significantly high risk of mortality, indicating that the levels of DAMPs may play an anti-inflammatory role, regulating the immune response [21].

Acquired immune system dysfunction characterized HD patients [22].

B and T lymphocytes balance the immune system by the mutual restriction of CD4+ T helper and CD8+ T suppressor cells, cooperating with the innate immune cells [23]. CD4+ cells, isolated by HD patients, are characterized by a reduced expression of key surface antigens, altering the function of B lymphocytes, which largely depend on their activation [24].

The reasons for these quantitative and qualitative abnormalities are still unclear and could be related to multiple pathways, including the accumulation of uremic toxins [25]. Free light chains (FLCs) may be markers of inflammation and immunity dysfunction.

Two isotypes (monomeric κ and dimeric λ) are produced in excess in chronic inflammation and, with molecular masses of 22.5 and 45 kDa, respectively, accumulate in HD patients [26,27,28]. In functional kidneys, serum (s) FLCs are primarily removed by the catabolism in the proximal tubular, determining serum κFLCs concentrations lower than λFLCs concentrations, with a median ratio of 0.58 (normal range 0.26 to 1.65) [29]. During the progression of nephropathy, sFLC levels will saturate the metabolic capacity of the kidney, and only after this point will later be detectable in the urine. In severe renal failure, the reticuloendothelial system becomes the main route of their removal, and the serum half-life of FLCs increases to 32 h or more [30].

Whereas several studies have assessed the relationship between FLCs and mortality risk in chronic kidney disease (CKD) [31,32,33], FLCs received only marginal attention as uremic toxins in non-multiple myeloma HD patients. Lamy evaluated their reduction with different dialyzers, revealing a better removal of κFLCs, but not λFLCs, after hemodiafiltration (HDF) if compared to bicarbonate dialysis [34]; expanded HD (HDx), based on medium cut-off (MCO) dialyzers, recently demonstrated non-inferiority results about FLC removal, when compared with HDF data [35,36].

This prospective study aimed to evaluate the clinical impact of FLCs in HD patients, analyzing their relations with other biomarkers of inflammation and immunity status, such as C-reactive protein (CRP), serum HMGB1, and main lymphocyte subsets. Furthermore, we assessed the role of these biomarkers on mortality risk.

## 2. Materials and Methods

### 2.1. Study Design and Population

This study is a single-center prospective cohort study evaluating the association between FLC and adverse outcomes in adults treated with long-term hemodialysis.

We enrolled one hundred and nineteen HD patients at the Nephrology and Dialysis Unit of Papardo Hospital in Messina, Italy, between March 2018 and March 2022. Inclusion criteria were age > 18 years, absence or <200 mL/die residual diuresis, fistula or central venous catheter with blood flow > 250 mL/min. We included only patients with a κ/λ ratio within the renal reference range (0.37–3.1).

We excluded from the analysis patients with paraproteinemia, defined as abnormal FLC (κ/λ) ratio using the renal reference range (0.37–3.1), an elevation of the involved light chain and positive serum protein electrophoresis, and immunofixation result [31]. Other exclusion criteria were cancer, active viral infections, history of transplantation, immunosuppressive treatments, or a recent infectious episode (<3 months).

Patients were included at least six months after the onset of renal replacement therapy, receiving three-weekly HD sessions lasting 4 h. The dialytic regimen and prescription were maintained stable for six months before the enrollment and during the entire study period.

All demographic, clinical, dialytic, and laboratory data were collected during the enrollment period by the nephrologists of the Centre.

The primary outcome was four years all-cause mortality. Study patients were followed until death or until the end of the study in March 2022. All patients were previously informed and gave their written consent. The University of Messina Ethics Committee approved the study (approval number n°20/20), and all procedures were in accordance with the Declaration of Helsinki.

### 2.2. Laboratory Analyses

We collected blood samples before the start of the first dialysis session of the week. The serum was separated in a refrigerated centrifuge and then stored at −80 °C until analysis for κFLCs, λFLCs, and HMGB1. For data analysis, κFLC and λFLC were summated to give a combined (c) FLCs concentration. The reference range of normal cFLC levels was 9.3–43.3 mg/L [37]. Flow cytometry assessed CD3+, CD4+, and CD8+ lymphocyte subsets.

### 2.3. Statistical Analyses

Statistical analyses were performed with MedCalc and GraphPad Prism software. Data were presented as mean ± SD, median (range), or percentage frequency as appropriate. Differences between groups were established by unpaired *t*-test for normally distributed values and by Kruskal–Wallis analyses, followed by Dunn’s test for nonparametric values. Pearson or Spearman correlation coefficients were used to test correlations between cFLCs and other variables. All non-normally distributed values were log-transformed to better approximate normal distributions. To find the best cut-off values for identifying the progression to the endpoint, receiver operating characteristics (ROC) analysis calculated the area under the curve (AUC) for cFLCs and other markers. Kaplan–Meier curves assessed survival in subjects with cFLCs and sHMGB1 values above and below the optimal ROC-derived cut-off levels. Cox proportional hazard regression analyses calculated adjusted risk estimates for the progression to the endpoint. All results were considered significant if *p* was <0.05.

## 3. Results

### 3.1. Baseline Characteristics

Table 1 summarizes the baseline data.

The population had a median age of 71 years (IQ = 55.2–76.7), with a mean dialysis vintage of 57.6 ± 16.8 months. The mean dialysis session length was 240 ± 0.11 min, and the mean values of single-pool KT/V were 1.4 ± 0.3.

Diabetic nephropathy represented the primary renal disease in 58 patients (49%); hypertensive nephrosclerosis was detected in 34 subjects (29%), whereas chronic glomerulonephritis and polycystic kidney disease were detected in 16 (13%) and 11 (9%) patients, respectively.

The artero-venous fistula represented vascular access in ninety-five patients, whereas the remaining 20% had a central venous catheter. Twenty-eight patients (24%) underwent HD with high-flux polysulfone (Fx60, Fresenius, Oberrursel, Germany), whereas thirty-six (30%) patients were treated with acetate-free biofiltration (AFB) with polyacrylonitrile (AN69ST; Baxter, Medolla, Italy). Furthermore, forty patients (34%) underwent online hemodiafiltration (HDF) using the high-flux polysulfone Fx1000 filter (Fresenius, Oberrursel, Germany). Fifteen patients (12%) were treated with expanded hemodialysis (HDx) using a medium cut-off filter (Theranova, Baxter, Medolla, Italy).

### 3.2. FLC Levels

**Figure 1:**
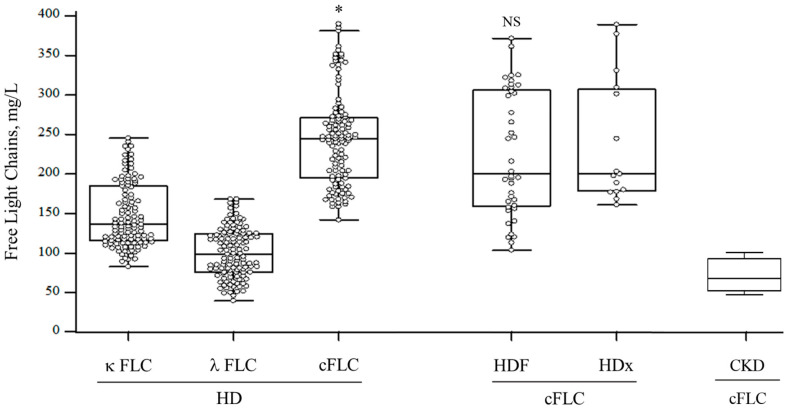
Free light chain values of the studied cohort, Abbreviations: HD: hemodialysis; cFLC: combined free light chain; HDF: hemodiafiltration; HDx: expanded hemodialysis; CKD: chronic kidney disease; *: *p* < 0.001 differences of cFLC in HD vs. CKD patients. NS: *p* > 0.05 differences of cFLC in HDF vs. HDx group.

High FLC levels characterized HD patients. κFLC values were 137.9 (115.5–190.4) mg/L, λFLC levels were 99.3 (76.4–124) mg/L, whereas cFLCs were 244.4 (197.9–273.5) mg/L. These values were extremely higher than those characterizing healthy subjects (median = 28 mg/L; normal range = 9.3–43.3 mg/L) [37], and CKD patients [68.9 mg/L (49.4–100.9); *p* < 0.001] with an estimated glomerular filtration rate of 30 (21–41) ml/min [38]. The κ/λ FLCs ratio assessed in our cohort was 1.71 (1.5–2.5). We did not observe statistical differences in cFLC levels according to dialysis techniques. The bicarbonate HD group was characterized by 207.7 (185.1–289.6) mg/L of cFLC levels with similar median values assessed in HDF [195.2 (154.7–325.8) mg/L; *p*: 0.35], AFB [194.3 (168.8–319.7) mg/L; *p*: 0.08] and HDx [202.9 (178–331. 6) mg/L; *p*: 0.10] (Figure 1).

### 3.3. Inflammatory and Immunologic Markers

CRP levels were 0.5 (0.5–1.4) mg/dL, whereas procalcitonin (PCT) values were 0.2 (0.1–0.3) ng/mL. We also tested acute-phase reactant proteins, such as α1 (4.9 ± 1.1 mg/dL) and α2 (10.2 ± 2 mg/dL). Serum β2-microglobulin (β2-MG) levels were 28.7 (22.1–32.4) mg/L.

We investigated some markers of the acquired immune system. WBC count was 7.070 ± 2.460 mm^3^, with 64.4 ± 12.9% of neutrophils and 20 ± 5.7% of lymphocytes. The neutrophil/lymphocyte ratio was 3.6 ± 1.7. The mean values of CD3+ cells were 72.7 ± 11.9, CD4+ cells were 42.1 ± 11.7, and the median value of CD8+ cells was 28 (21–34). We detected a significant reduction in CD8+ cells and a decreased ratio of CD4+/CD8+ if compared with the healthy control group [1.7 (1.4–1.9), *p*: 0.02]. The median value of the CD4+/CD8+ ratio was 1.1 (0.7–1.5), with a ratio < 1 observed in 31 patients. We evaluated the variation of these subtypes of cells during a single dialysis session.

We revealed a reduction in CD8+ cells at the end of a single session [493 (256–619.5) vs. 360 (219.5–505) count; *p*: 0.001], without variations in the percentage of CD3+ and CD4+ cells.

**Figure 2:**
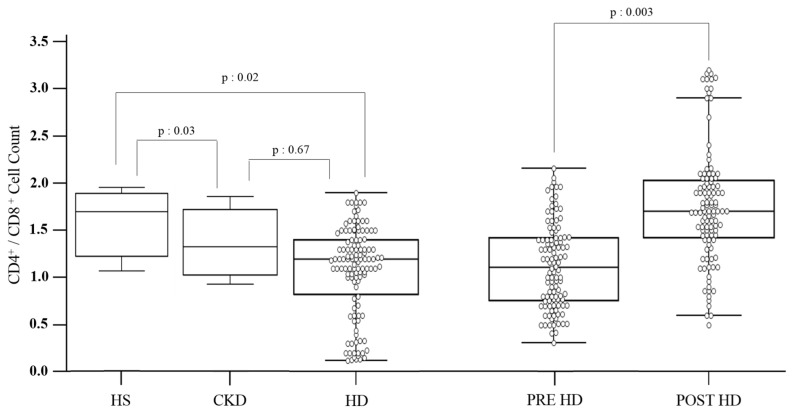
CD4+/CD8+ ratio values of the studied cohort.

We assessed an increased CD4+/CD8+ ratio immediately after the end of the dialysis if compared with the pre-dialytic values [1.7 (1.2–2) vs. 1.1 (0.7–1.5); *p*: 0.003] (Figure 2).

There was no significant difference in CD3+, CD4+, CD8+ levels, and CD4+/CD8+ ratio among different HD techniques (*p* > 0.05). We revealed variable levels of HMGB1 [(94.5 (55–302) pg/mL], with a wide fluctuation of its values among HD patients.

### 3.4. Correlates of cFLCs

On univariate analysis, cFLCs, on a natural logarithmic scale (LogFLCs), positively correlated with β2-MG (r = 0.50; *p* < 0.0001), hemoglobin (r = 0.22; *p* = 0.02), total serum protein (r = 0.28; *p* = 0.002), and gamma globulins (r = 0.31; *p* = 0.0006). An inverse correlation has been revealed with CRP (r= −0.31; *p* < 0.001), PCT (r = −0.22; *p* = 0.01), ferritin (r = −0.31; *p* < 0.001), alpha-1 globulins (r= −0.36; *p* < 0.001), and CD4+/CD8+ ratio (r= −0.28; *p* < 0.001).

Using cFLCs as the dependent variable in a multiple regression model, including all previously reported univariate correlates, the associations with β2-MG (β = 0.40, *p* = 0.003) and CD4+/CD8+ ratio (β = −0.31; *p* = 0.001) remained significant.

### 3.5. Mortality in HD Patients

Thirty-six patients (30%) died during follow-up (progressors), with a median survival time of 16.3 ± 11.7 months (IQR = 5–26.7). The remaining eighty-three patients (70%; non-progressors) completed the observational period. Table 1 displays the data and statistical differences between progressors and non-progressors.

Progressors presented increased cFLC and β2-MG values and low CD4+/CD8+ ratio and HMGB1 levels at baseline.

**Figure 3:**
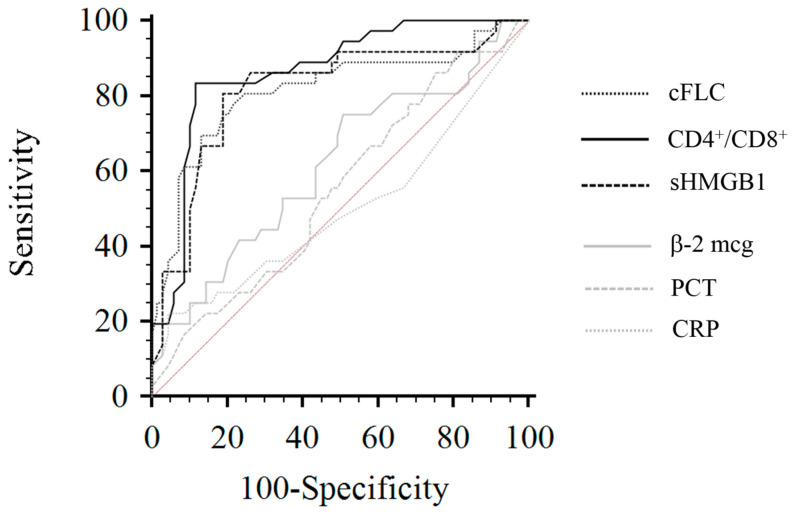
Receiver operating characteristics curves of cFLC, sHMGB1, CD4+/CD8+ ratio, CRP, β2-MG, and PCT considering mortality as status variable. Abbreviations: cFLC: combined free light chain; sHMGB1: serum high mobility group box 1; CRP: C-reactive protein; β2-MG: β2-microglobulin; PCT: procalcitonin.

ROC analysis showed an AUC for cFLC, CD4+/CD8+ ratio, and sHMGB1 of 0.81 (95% CI, 0.72–0.88), 0.86 (95% CI, 0.78–0.92), and 0.83 (95% CI, 0.78–0.89), respectively. cFLC area was statistically different from β2-MG (*p*:0.006). Similarly, the CD4+/CD8+ ratio and sHMGB1 areas highlighted better diagnostic profiles in terms of sensitivity and specificity than PCT and CRP (*p* < 0.001). For cFLCs, the best cut-off level was 263 mg/L (sensitivity 75.7%, specificity 80%), whereas for sHMGB1 and CD4+/CD8+ ratio it was <80 ng/mL (sensitivity 81.6%, specificity 80.5%) and <1 (sensitivity 81.6%, specificity 88.9%), respectively. Figure 3 shows reports from the ROC analysis.

**Figure 4:**
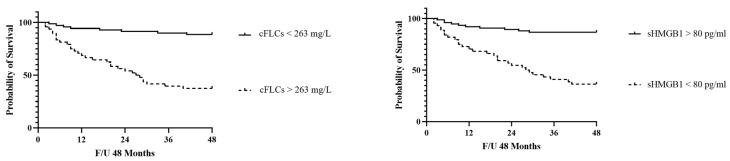
Kaplan–Meier survival curves of endpoint in patients with combined free light chain (cFLC) and serum high mobility group box 1 (sHMGB1) levels above and below the optimal receiver operating characteristics cut-off levels of 163 ng/L and 80 pg/mL.

Figure 4 presents Kaplan–Meier survival curves of patients with cFLC and sHMGB1 levels above and below the optimal cut-off; subjects with cFLC values above 263 mg/L experienced a significantly faster evolution to the endpoint (*p* < 0.001), with a mean follow-up time to progression of 27.5 months (95% CI, 4.2–16.5). A similar evolution has been noted in patients with sHMGB1 values < 80 pg/mL (*p* < 0.001; mean follow-up time to progression of 28.5 months). The combination of the two markers (cFLCs > 263 mg/L associated with sHMGB < 80 pg/mL) did not worsen the percent of patient survival if compared to the previous analyses (*p*: 0.67).

### 3.6. Univariate/Multiple Cox Regression Analysis and Mortality Risk in HD Patients

To identify putative risk factors associated with death, we conducted a Cox regression analysis, inserting in the model all variables that were different at baseline in patients who reached the endpoint during the follow-up period. At univariate analysis, BNP, β2-MG, cFLCs, and sHMGB1 were significantly associated with the endpoint, whereas diabetes, ferritin, CRP, CD4+/CD8+ ratio, and age failed to reach statistical significance. We performed a multiple Cox regression, simultaneously inserting into the model all the variables significantly associated with the endpoint at univariate analysis. Age was also inserted in this model, although it was not associated with the endpoint. Results from this analysis indicated that both cFLCs and sHMGB1 predicted a higher risk of mortality independently from BNP and β2-MG. In detail, cFLCs were associated with an 11% increased risk of death (HR 1.11; 95% CI, 1.06–1.13; *p*: 0.02), whereas low sHMGB1 increased this risk by 5% (HR 0.95; 95%CI, 0.89–0.98; *p*: 0.03). Table 2 summarizes data from Cox analyses.

## 4. Discussion

To the best of our knowledge, this is the first prospective study revealing the association between cFLCs, HMGB1, and mortality in HD patients.

Elevated FLC levels characterized our cohort, with median values higher than those observed in CKD patients, due to abnormal production and an inadequate clearance by hemodialysis. Whereas Cohen demonstrated that bicarbonate dialysis and HDF were unable to normalize FLC values [39], in the last years, growing data highlighted a better clearance of medium molecules, including FLCs, through HDF with high convective volumes and HDx. Moreover, dialyzer performance significantly affected 3-year mortality, revealing that MCO filters improved mortality outcomes [40].

Whereas in CKD patients the impact of FLCs on mortality is still controversial [6,37], the excessive FLC endocytosis by proximal tubular cells and their accumulation at the distal tubule represent the main processes of the progression of the renal disease, with inflammation and pro-fibrotic effects [41,42,43].

However, FLCs are not only simple markers of inflammation such as CRP or PCT. Interestingly, FLCs are inversely correlated with CRP, PCT, and alpha-1 globulins. The kinetics of CRP and cFLC levels differ, with CRP levels more closely associated with acute, but not chronic, inflammation [44]. Similarly, PCT levels rise 3 to 6 h after a bacterial infection or sepsis, without significant variations in patients with non-infectious inflammation. Moreover, according to our ROC data, PCT and CRP revealed weaker diagnostic information about our endpoint than cFLCs, with low sensitivity and specificity. Another uremic toxin, β2-MG, had a better diagnostic profile and was positively correlated with cFLCs after multivariate analysis.

We assessed that this toxin represents an independent marker of mortality in our HD cohort, strengthening well-known data available in the literature [45,46] and considering β2-MG as another actor of inflammation and immune dysfunction in the uremic population.

Our data demonstrate the central role of these middle molecules, revealing a complex process growing during several years of pre-dialytic CKD, and achieving the peak during the dialysis period. This process has a common denominator: a vicious cycle between sub-clinical, chronic inflammation and quantitative and qualitative immune dysfunctions.

FLCs can modulate the qualitative functions of polymorphonuclear leukocytes by inhibiting spontaneous apoptosis and decreasing chemotaxis and glucose uptake [4]. The decreased granulocyte and monocyte/macrophage phagocytic function and the reduced capacity of antigen-presenting cells represent the main processes of natural immune dysfunction in these patients [25].

However, in clinical practice, few biomarkers adequately identify innate and acquired immunity dysfunction in HD patients.

We assessed the role of HMGB1 as one of the markers of the innate immune system, revealing higher values than those observed in CKD. Acute inflammatory and infective processes, such as acute kidney injury and sepsis, determine high HMGB1 levels [21]. Hypoxic, injured, or dying cells release DAMPs, activating the immune system and promoting inflammation [47,48]. According to these data, high HMGB1 values observed in our patients reflect the permanent, active inflammation and the consequent reactive response of the natural immune system. We assessed, interestingly, low levels of HMGB1 in patients who died during the follow-up period, demonstrating, after multivariate Cox analysis, that this alarmin represents an independent risk factor of mortality in our cohort.

Previous data revealed higher serum levels of this peptide in HD subjects if compared with CKD patients or those treated by peritoneal dialysis, with a time-dependent manner reduction [17]. Our data corroborate these findings, suggesting that reduced levels of HMGB1, characterizing our inflamed patients, are associated with a concomitant chronic depletion of innate immune cells, the leading source of this alarmin. If this process occurs, it has obvious consequences in terms of mortality risk.

In addition to the innate immune system, an altered acquired immunity characterizes HD patients. T lymphocyte dysfunction, found in ESRD, can be attributed to impaired innate immunity and dysfunction of Toll-like receptors, whose HMGB1 represents the leading ligand [49,50], associated with an almost linear decrease in the total B cell count, CD4+, and the CD8+ T cell compartment [51].

We detected a significant reduction in the percentage of CD8+ cells and a decreased CD4+/CD8+ ratio; the latter increased after a single dialysis session, suggesting that HD can temporarily improve this immune system. However, the accumulation of uremic toxins during the inter-dialytic period negatively and gradually acts on cellular function, as confirmed by the inverse relationship found in our cohort, after multivariate analysis, between FLCs and CD4+/CD8+ ratio. This datum links high inflammation to immune depression, such as the low CD4+/CD8+ ratio, characterizing patients who died during the follow-up and mirroring a suppressed acquired immunity. Our results were consistent with previous studies, indicating exhaustion of acquired immunity in HD patients due to a decrease in circulating naive T cells and age-related changes related to the pro-inflammatory environment, named in flammageing, observed in the uremic population [52].

However, all these markers could only partially highlight the immune dysfunction occurring in HD patients, with the necessity of further studies to corroborate these results and to create a panel of biomarkers evaluating all the immunologic pathways altered in these patients.

Nevertheless, if the subclinical inflammatory process involves all HD patients, the same patients could be widely heterogeneous from an immunological point of view, as suggested by the high variability of some immune markers, supposing different immune profiles among dialyzed patients. The clinical implications are that specific immune profiles may identify an increased risk of acute rejection, evaluable before transplantation, or may favor viral or bacterial infections, cause poor response to vaccinations, or increase the risk of malignancies. Betjes’ results support our hypothesis, revealing that patients with a higher frequency of terminally differentiated CD8+ cells had a decreased risk of acute rejection [53].

The present study has some limitations.

First, it was a single-center study, and the cohort of patients was relatively small. These limitations did not allow us, for example, to evaluate the influence of various dialytic techniques, the different causes of death, or immune profiles on FLCs or HMGB1. Confirmation in larger cohorts is indispensable to attribute general validity to our reports.

In the progressors group, the mean age was higher, with more patients with diabetes and in hemodialysis therapy for a longer time. One-third of the participants reached the endpoint during the follow-up, and the statistical model was powerful enough to establish independent relationships between cFLCs, HMGB1, and death.

Further in-depth examinations should verify whether these findings could be confirmed in a long-term observational period, determining if therapeutic measures targeting cFLCs and immune markers can improve HD patient survival. In clinical practice, our results might suggest stratifying HD patients according to FLCs and HMGB1 levels, personalizing the dialytic prescription with potential benefits from diffusive–convective methods and HDx techniques, and identifying patients with high mortality risk.

## 5. Conclusions

cFLCs and HMGB1 represent two independent risk markers of mortality in hemodialysis patients.

## Figures and Tables

**Table 1:** Baseline demographic, clinical, and laboratory data of the study population.

Variable	All Patients (n = 119)	Progressors (n = 36)	Non Progressors (n = 83)	*p*
Age, years	71 (55.2–76.7)	75.7 (68.5–78.9)	60.2 (53.2–65.8)	**<0.01**
M/F	82/37	21/15	61/22	0.13
Dialysis vintage, months	57.6 ± 16.8	63.2± 11.3	58.1± 13.4	0.09
spKt/V, weekly mean	1.4 ± 0.3	1.4 ± 0.2	1.3 ± 0.1	0.37
Dialysis session length, min	240 ±0.11	240 ± 0.14	240 ± 0.10	0.47
Diabetes, n (%)	67 (56)	31(86)	36 (43)	**0.01**
Hypertension, n (%)	61 (51)	21	40	0.32
**Laboratory parameters**				
Creatinine, mg/dL	9.73 ± 2.8	9.12 ± 1.8	10.1 ± 1.2	0.67
Urea, mg/dL	167.2 ± 41.1	212.7 ± 23.6	196.3 ± 30.1	0.57
Potassium, mmol/L	5 ± 0.8	5.6 ± 0.4	5.9 ± 0.2	0.89
Albumin, g/dL	3 ± 1.3	3.3 ± 0.9	3.9 ± 0.2	0.77
Phosphate, mg/dL	5.1 ± 1.8	6.2 ± 0.7	5.8 ± 1.1	0.61
Serum Calcium, mg/dL	8.4 ± 0.7	8.9 ± 0.3	9.1 ± 0.6	0.45
PTH, pg/mL, median (IQR)	321 (186–428)	387 (202–387)	341 (232–431)	0.09
Total cholesterol, mg/dL	140.5 ± 32.3	162.5 ± 22.1	151.1 ± 12.9	0.32
White blood cells, mm^3^	7.07 ± 2.4	8.12 ± 1.9	8.9 ± 1.1	0.23
Hemoglobin, g/dL	11.5 ± 1.2	10.7 ± 0.9	11.7 ± 0.7	0.09
TSAT, %, median (IQR)	27.8 (22.1–40.1)	23.1 (19.4–33.2)	28.1 (21.7–36.1)	0.11
BNP, pg/mL, median (IQR)	5440 (1650–9960)	10,457 (6594–12,660)	4679 (1369–7460)	**<0.01**
**Inflammatory markers**				
Ferritin, ng/mL median (IQR)	605.5 (448–914)	816 (723–1021)	493 (401–613)	**<0.01**
CRP, mg/dL median (IQR)	0.5 (0.5–1.4)	1.7 (1.4–2.1)	0.4 (0.2–0.9)	**0.02**
PCT, ng/mL median (IQR)	0.2 (0.1–0.3)	0.4 (0.2–0.5)	0.3 (0.2–0.4)	0.21
Homocistein, μmol/L	30.19 ± 13.9	33.5 ± 8.7	31.2 ± 11.4	0.30
α1 protein, mg/dL	4.9 ± 1.1	5.2 ± 0.7	4.7 ± 1.3	0.49
α2 protein, mg/dL	10.2 ± 2	9.7 ± 1.3	10.1 ± 1.2	0.87
β-2 MG, mg/L, median (IQR)	28.7 (22.1–32.4)	32.1 (28.5–36)	21.2 (18.5–28)	**0.02**
cFLC, mg/L, median (IQR)	244.4 (197.9–273.5)	251 (205–341)	177.5 (161–207)	**<0.01**
**Immunity markers**				
sHMGB1, pg/mL, median (IQR)	94.5 (55–302)	67 (54–111)	152 (98–297)	**<0.01**
White blood cells, mm^3^	7.07 ± 2.4	8.1 ± 1.9	8.9 ± 1.1	0.25
CD4+/CD8+ ratio, median (IQR)	1.1 (0.7–1.5)	0.7 (0.6–0.9)	1.2 (0.9–1.6)	**0.01**
γglobulin, UA/mL	15.1 ± 4.1	13.2 ± 1.8	14.9 ± 2.8	0.11

Values for categorical variables given as percentage; data are expressed as mean ± SD, or median (IQR) interquartile range (25th percentile, 75th percentile). Abbreviations: spKt/V, single-pool Kt/V; PTH: parathyroid hormone; TSAT: transferrin saturation; BNP; brain natriuretic peptide; CRP: C-reactive protein; PCT: procalcitonin; β-2MG: β-2 microglobulin; sHMGB1: serum high mobility group box 1; cFLC: combined free light chains.

**Table 2:** Univariate and multivariate Cox proportional hazards regression model for death during the follow-up period.

	Univariate Analysis			Multivariate Analysis		
	HR	95% CI	*p* Value	HR	95% CI	*p* Value
Age	1.03	0.91–1.06	0.11	1.03	0.97–1.04	0.16
Diabetes mellitus	1.10	0.95–1.21	0.32			
BNP	1.03	1.01–1.05	<0.01	1.08	1.04–1.10	0.01
Ferritin	1.06	0.97–1.18	0.37			
CRP	1.10	0.98–1.09	0.08			
β2-MG	1.09	1.01–1.13	<0.01	1.09	1.05–1.12	0.02
CD4+/CD8+ ratio	1.02	0.97–1.04	0.10			
sHMGB1	0.83	0.74–0.96	0.02	0.95	0.89–0.98	0.03
cFLCs	1.02	1.01–1.03	0.01	1.11	1.06–1.13	0.02

Abbreviations: BNP: brain natriuretic peptide; CRP: C-reactive protein; β2-MG: β2 microglobulin; sHMGB1: serum high mobility group box 1; cFLCs: combined free light chains.

## Data Availability

The dataset generated and analyzed during the current study is available from the corresponding author on reasonable request.
